# Supplementary material for: High-Intensity Interval Training is Associated with Improved Long-Term Survival in Heart Failure Patients
Source: J Clin Med. 2019 Mar 25;8(3):409. doi: 10.3390/jcm8030409 (PMC6462952; doi:10.3390/jcm8030409)
Supplement: Supplementary file 1 [file jcm-08-00409-s001.pdf]

Supplemental Content

Supplemental Data: Rebalancing results for Propensity score matching (PSM) is described in the following. The group HIIT and MDP shows marginal significant difference in their age. (61.5 versus 64.7 with p=0.07) These potential confounding factors could influence the interpretation of our cox regression analysis for HF patients' survivals. The PSM could be used to reduce the effects of these confounding variables. In this study, we perform simple PSM matching between HIIT and MDP groups on their age, sex and LVEFP. The final matched balance results are shown below. As you can see that after matched, the age difference was greatly reduced and gender distribution is equalized between male and female. Although the difference of LVEFP was reduced after matching, the significant difference still remains in our data. The low LVEFP in HIIT group will reduce the effectiveness of HIIT on the survival analysis since those patients are in poor conditions than those from control group. Lastly, it is important to note that our matching was carried out without replacement. Therefore, we are selecting those 101 controls out of total 133 patients from MDP group to match their corresponding HF patients from HIIT group.

```
***** (V1) AGE *****
                       Before Matching       After Matching
mean treatment........     61.455               61.455
mean control..........     64.729               62.792
std mean diff.........    -23.395              -9.5515

mean raw eQQ diff.....     3.2376               2.2475
med  raw eQQ diff.....         3                2
max  raw eQQ diff.....         9                9

mean eCDF diff........   0.058831             0.038871
med  eCDF diff........   0.061044             0.039604
max  eCDF diff........    0.12529             0.079208

var ratio (Tr/Co).....     1.0588               1.0539
T-test p-value........   0.074206              0.46723
KS Bootstrap p-value..       0.5                1
KS Naive p-value......    0.32839               0.9093
KS Statistic..........    0.12529             0.079208


***** (V2) SEX *****
                       Before Matching       After Matching
mean treatment........     1.3069               1.3069
mean control..........     1.2632               1.2673
std mean diff.........     9.4435               8.5442

mean raw eQQ diff.....   0.049505             0.039604
med  raw eQQ diff.....         0                0
max  raw eQQ diff.....         1                1

mean eCDF diff........   0.021886             0.019802
med  eCDF diff........   0.021886             0.019802
max  eCDF diff........   0.043773             0.039604

var ratio (Tr/Co).....     1.0997               1.0861
T-test p-value........    0.46625              0.52772
***** (V3) LVEF0 *****
                       Before Matching       After Matching
```

```
mean treatment........     34.259           34.259
mean control..........     42.812           37.026
std mean diff.........    -56.624          -18.315

mean raw eQQ diff.....     8.2851           2.7861
med  raw eQQ diff.....        7            2.2
max  raw eQQ diff.....      17.5             9

mean eCDF diff........    0.12423         0.042904
med  eCDF diff........   0.14446          0.039604
max  eCDF diff........   0.22266           0.12871

var ratio (Tr/Co).....    0.77885           1.0407
T-test p-value........ 7.0545e-05         0.010997
KS Bootstrap p-value.. < 2.22e-16             0.2
KS Naive p-value......  0.0067449          0.37279
KS Statistic..........    0.22266          0.12871


Before Matching Minimum p.value: < 2.22e-16
Variable Name(s): LVEF0  Number(s): 3

After Matching Minimum p.value: 0.010997
Variable Name(s): LVEFP0  Number(s): 3
```

Supplemental Figure S1: In FPCA data analysis, each individual longitudinal response of LVEF_Diff, normalized VO_{2peak}_Diff, normalized LVEDD_Diff or normalized LVESD_Diff were modeled by eigenfunctions and are selected until 95% of variances are explained by the model

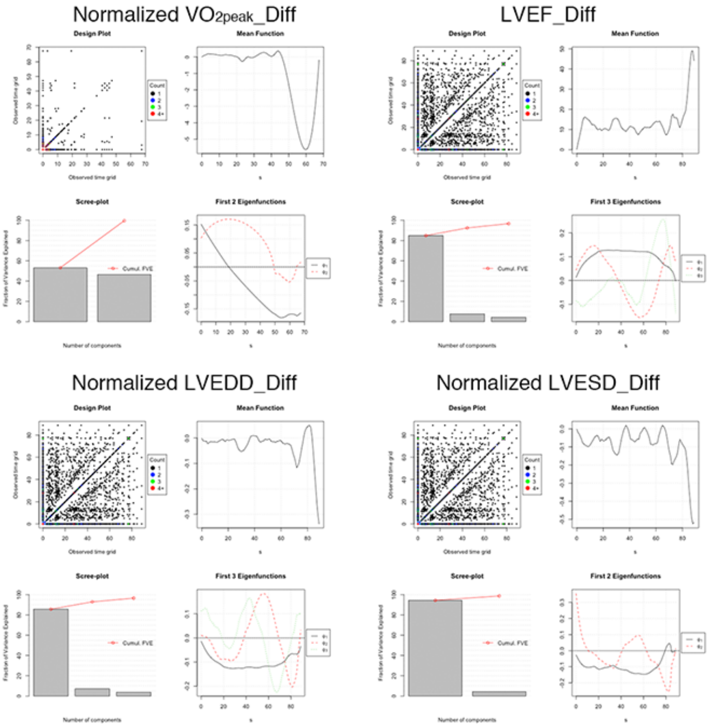

Supplemental Figure S2: Landmark analysis for cumulative mortality events. The landmark time was set at one year (······). Although the difference between the exercise (—) and non-exercise (---) participants was not significant, the increased trend of cumulative mortality was observed in non-exercise participants in the first and the 8th follow-up years.

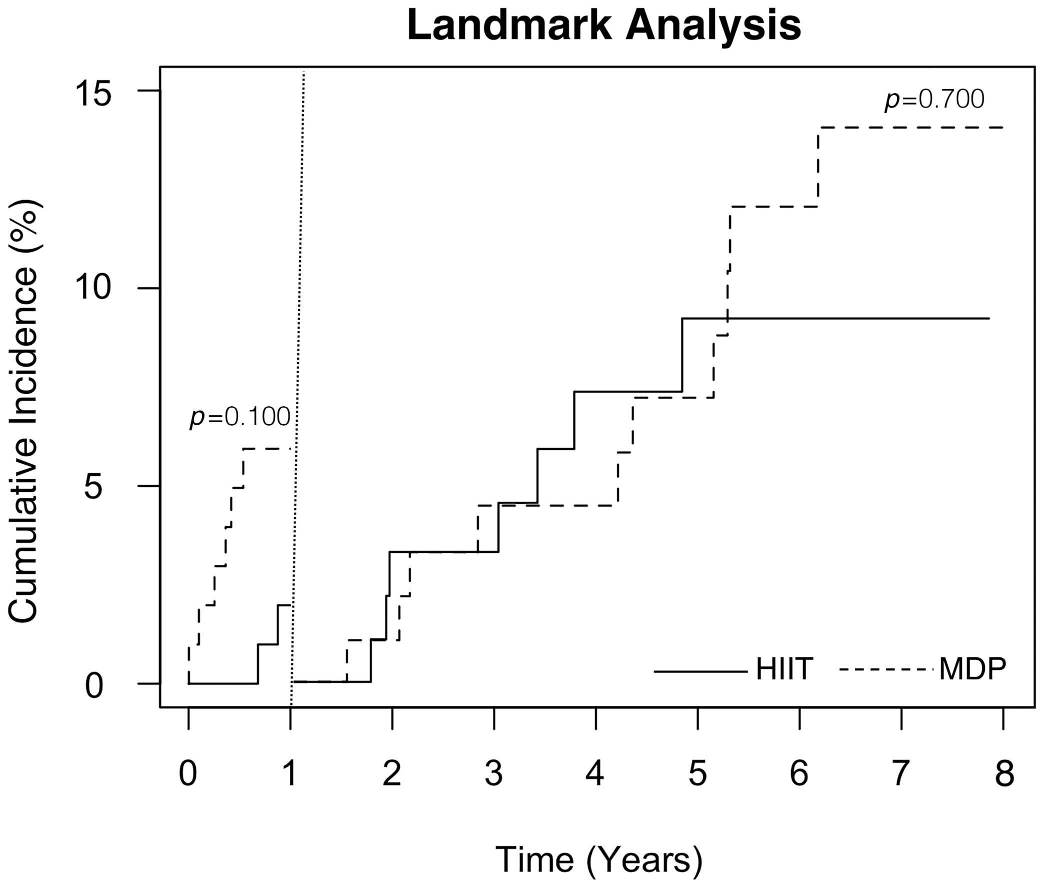

Supplemental Figure S3: Different stress responses to HIIT between HFrEF (gray) and HFpEF (white) patients.105 HFrEF patients (58 HIIT and 47 MDP participants) and 41 HFpEF patients (21 HIIT and 13 MDP participants) had baseline b-type natriuretic peptide (BNP) levels. Subsequent evaluations were done in 43 HFrEF (24 HIIT and 19 MDP participants) and 15 HFpEF patients (6 HIIT and 9 MDP participants). We observed that HIIT induced a significant decrease ($p < 0.001$) of BNP (mean (95% CI)) from 518 (564–936) pg/mL to 92.8 (86–222) pg/mL in HFrEF patients but no significant changes detected in HFpEF patients. The bar represented median with 1st-3rd quartiles and standard error covered the minimum to the maximum values. Outliers were shown in dots.

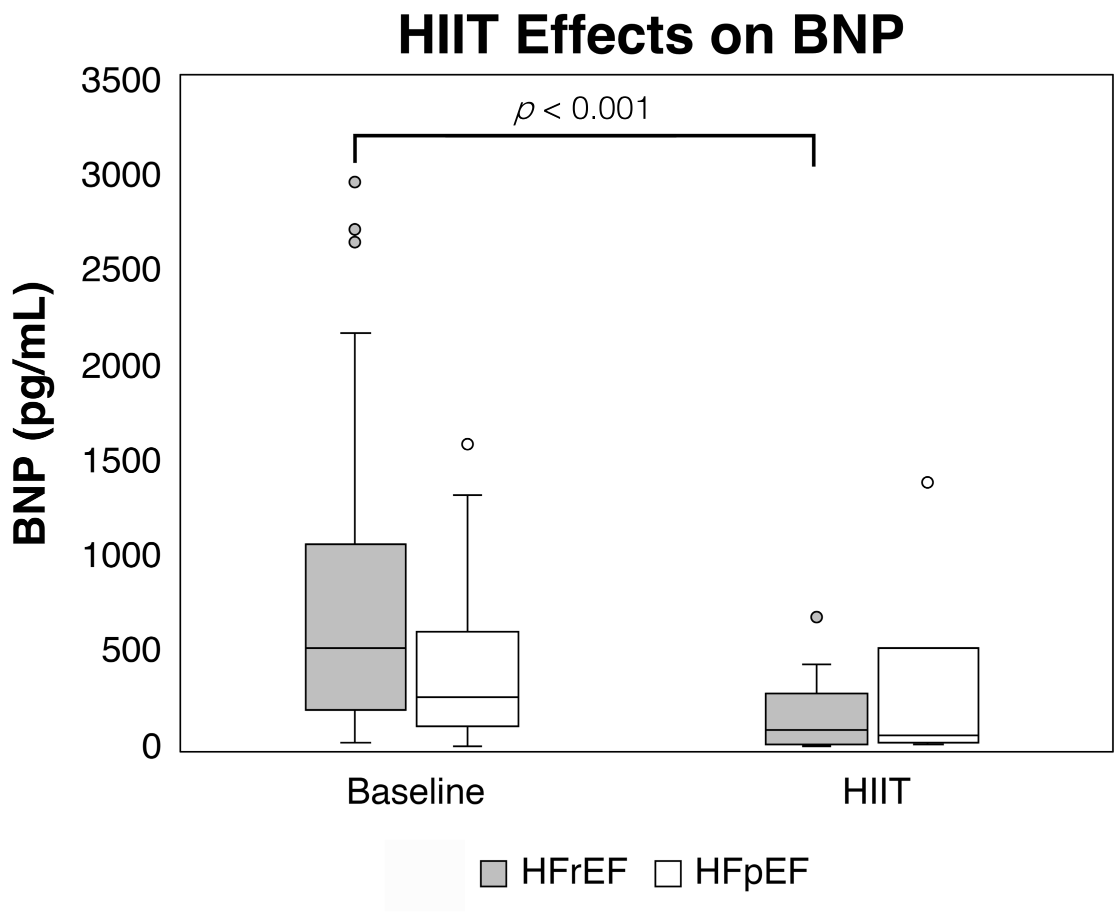

Supplemental Table S1: List of all variables in the study

Diagnosis	1.CHF // 2.CAD (2-1Angina / 2-2. Unstable Angina / 2-3.AMI / 2- 4. STEMI / 2-5. NSTEMI / 2-6. old MI / 2-7.LV thrombus / 2-8. HCVD / 2-9. A.A.A / 2-10.VSD / 2-11.ASD / 2-12. aortic dissection // 3.DCM // 4. VHD (4-1.MS / 4-2. MR / 4-3.AS/AR / 4-4.MVP s/p tissue MVR) // 5. Arrhythmia (5-1. Af / 5-2. VT / 5-3. CRBBB / 5-4. AV block / 5-5. S.S.S / 5-6.VPC / 5-7.Vf / 5-8.bradycardia) // 6.PAOD // 7. s/p CABG // 8. Stroke // 9. Hyperlipidemia (9-1. Dyslipidemia / 9-2. Hypercholesterolemia) // 10.DM // 11.HTN // 12. COPD (12-1. Asthma / 12-2. Pneumoconiosis) // 13. renal disease (13-1. ESRD / 13-2. CKD / 13-3. CRF / 13-4. ARF / 13-5. acute kidney injury) // 14. Alcoholism // 15. Gout (hyperuricemia)// 16. BPH // 17. SLE (Lupus nephritis) // 18. Cancer (18-1. Breast Cancer / 18-2. Rectal cancer / 18-3. Endometrioid Ca / 18-4. bronchioloalveolar carcinoma / 18-5. Cervical cancer / 18-6. Lung cancer / 18-7. Gastric cancer / 18-8. Colon adeno ca / 18-9. HCC 18-9. bladder cancer) // 19. Gastroenterology (19-1.GU / 19-2. PU / 19-3. DU / 19-4. GERD / 19-5.UGI bleeding / 19-6.Pancreatits) // 20. Infection (20-1. Pneumonia / 20-2. Urosepsis / 20-3. Cellulitis / 20-4. bronchitis / 20-5. Infective endocarditis / 20-6. Bronchopneumonia / 20-7. Bacteremia) // 21. Other (21-1. poliomyelitis / 21-2. Hyperthyroidism / 21-3. Hypothyroidism/ 21-4. anemia / 21-5. Thyroidectomy / 21-6. Hypoalbuminemia / 21-7. Postural hypotension / 21-8. Hematuria / 21-9. nodular goiter / 21-10. Scoliosis / 21-11. Pericarditis / 21-12. DVT /21-13. Thyroid storm / 21-14. HIVD / 21-15. Maculopapular rashes) // 22. Electrolyte imbalance (22-1. Hyponatremia / 22-2. Hypokalemia) // 23. OSA (Sleep apnea) // 24. Liver disease (24-1. Hepatitis / 24-2. liver cirrhosis / 24-3. abnormal liver function / 24-4. Fatty liver / 24-5. Alcoholic liver disease)
Whole ID	Chart Number
SEX	Female:0/Male:1
BIRTHDAY	yyyymmdd
Admission Date	yyyymmdd
Death Date	yyyymmdd
AGE	y
Death cause	Code as the diagnosis

Supplemental Table S2: Baseline blood chemistry in enrolled HF patients

	HIIT+MDP *n* = 101	MDP *n* = 101	*p*- Value
Cre, mg/dL[a]	1.12 (1.03-1.21)	1.39 (1.03-1.75)	0.158
LDL, mg/dL[b]	118 (107-129)	114 (106-123)	0.604
Hba1c, %[c]	6.51 (6.22-6.78)	6.90 (6.46-7.34)	0.143

Values are mean (95% CI).

Abbreviations: Cre, creatinine; Hba1c, glycohemoglobin; HIIT, high-intensity interval training; LDL, low-density lipoprotein; MDP, multidisciplinary disease management program.

[a] Baseline Cre levels in 98 subjects in exercise and 99 subjects in non-exercise groups.

[b] Baseline LDL levels in 97 subjects in exercise and 95 subjects in non-exercise groups.

[c] Baseline Hba1c levels in 88 subjects in exercise and 79 subjects in non-exercise groups.

Supplemental Table S3: Characteristics of deceased HF patients and causes of death during F/U.

		HIIT+MDP	MDP	*p*- Value
HFrEF/HFpEF		7/2	8/8	0.229
Sex (F/M)		1/8	2/12	0.355
Age, years		70.6 (58.2-82.9)	69.7 (62.4-77.0)	0.718
BMI, kg/m^2		23.0 (20.5-25.4)	26.3 (23.7-29.0)	0.074
F/U Period, month		38.3 (23.6-53.0)	30.8 (16.5-45.1)	0.452
VO_{2peak}, ml/kg/min		15.0 (13.1-16.9)	15.4 (12.6-18.1)	1.000
LVEF, %		41.4 (28.9-54.0)	41.5 (35.5-47.4)	0.934
LVEDD, mm		68.9 (63.1-74.7)	57.8 (54.7-61.0)	0.002[a]
LVESD, mm		54.2 (46.7-61.8)	45.8 (41.9-49.6)	0.065
SF-36	PCS	43.9 (33.8-54.0)	42.5 (37.7-47.4)	0.272
	MCS	47.3 (36.3-58.3)	41.9 (33.6-50.2)	0.446
Cause of death, *n* (%)				
Cardiac				
	HF	1 (11.1)	1 (6.3)	1.000
	Arrhythmia	3 (33.4)	5(31.0)	1.000
Non-cardiac				
	RF	–	1 (6.3)	1.000
	Infection	2 (22.2)	1 (6.3)	0.530
	Thrombocytopenia	1(11.1)	–	0.360
	Stroke	–	2 (12.5)	0.520
	Cancer-related	–	1 (6.3)	1.000
	GI	1 (11.1)	1 (6.3)	1.000
	Unknown	1 (11.1)	4 (25)	0.621

Values are mean (95% CI) or n (%).

Abbreviations: GI, gastro-intestinal disorder; RF, respiratory failure.

[a]Clinical information was assessed by Mann-Whiney U test.
